# Impact of Horizontal Edge–Interior and Vertical Canopy–Understory Gradients on the Abundance and Diversity of Bark and Woodboring Beetles in Survey Traps

**DOI:** 10.3390/insects11090573

**Published:** 2020-08-26

**Authors:** Jon Sweeney, Cory Hughes, Vincent Webster, Chantelle Kostanowicz, Reginald Webster, Peter Mayo, Jeremy D. Allison

**Affiliations:** 1 Natural Resources Canada-Canadian Forest Service, Atlantic Forestry Centre, 1350 Regent Street, P.O. Box 4000, Fredericton, NB E3B 5P7, Canada; cory.hughes@canada.ca (C.H.); vwebster2503@gmail.com (V.W.); chantelle.kostanowicz@canada.ca (C.K.); peter.mayo@canada.ca (P.M.); 2 24 Mill Stream Dr., Charters Settlement, NB E3C 1X1, Canada; reginaldwebster@rogers.com; 3 Natural Resources Canada-Canadian Forest Service, Great Lakes Forestry Centre, 1219 Queen Street E, Sault Ste. Marie, ON P6A 2E5, Canada; jeremy.allison@canada.ca; 4 Department of Zoology and Entomology, Forestry and Agricultural Biotechnology Institute, University of Pretoria, Pretoria 0002, Gauteng, South Africa

**Keywords:** survey and detection, trap placement, vertical gradient, horizontal gradient, Buprestidae, Cerambycidae, Dryophthoridae, Curculionidae, Scolytinae

## Abstract

**Simple Summary:**

Traps baited with sex attractants and plant odors are used by regulatory agencies to survey for alien invasive forest insects that may arrive via importation of goods from overseas. The performance of these surveys is affected not only by the type of traps and attractants used, but also by where the traps are placed at survey sites. We tested the effect of trap position along horizontal (relative to the forest edge) and vertical (canopy-understory) forest gradients on the diversity and abundance of species of bark and wood boring beetles detected. Both horizontal and vertical trap position affected trap performance, but trends differed among taxa and were context-dependent. For example, jewel beetles were detected mainly in canopy traps regardless of horizontal position, whereas bark and ambrosia beetles were detected mainly in understory traps placed along the forest edge. For optimal early detection of potentially invasive bark and wood boring beetles, surveys should place traps at multiple locations along horizontal and vertical gradients.

**Abstract:**

Semiochemical-baited intercept traps are important tools used to collect information about the presence/absence and population dynamics of forest insects. The performance of these tools is influenced by trap location along both horizontal edge–interior and vertical understory–canopy gradients. Consequently, the development of survey and detection programs requires both the development of effective traps and semiochemical lures but also deployment protocols to guide their use. We used field trapping experiments to examine the impact of both horizontal edge–interior and vertical understory–canopy gradients and their interactions with the species richness and abundance of Buprestidae, Cerambycidae and Curculionidae. Both gradients had significant effects on the diversity and abundance of all three families collected in traps and the pattern of gradient effects differed between the two experiments. In the first experiment, traps were deployed along transects involving large (>100 m) forest gaps and in the second experiment traps transected small (ca. 15 m) forest gaps. These results were consistent with the idea that gradient effects on the abundance and diversity of these three families of forest Coleoptera are context dependent. The results of this study suggest that monitoring programs for bark and woodboring beetles should deploy traps at multiple locations along both vertical understory–canopy and horizontal edge–interior gradients.

## 1. Introduction

Protocols which guide decisions about the need for management action are a central, defining element of integrated pest management (IPM) programs. These protocols are based on central decision rules which usually involve: (i) the assessment of the density of the pest population; (ii) an economic threshold; and (iii) phenological forecasting to determine the appropriate time to assess pest density [1]. This information is the basis of decision making in IPM.

The assessment of the pest population density requires the development of sampling programs which involve two components, the sampling technique(s) and deployment protocol which guide the use of the sampling technique [2]. Numerous sampling techniques exist for forest insects. In general, active techniques (e.g., trap trees, semiochemical-baited traps) are more specific than passive ones (e.g., unbaited Malaise and flight intercept traps), although there is some bias inherent in all sampling techniques. As a result, active sampling programs are easier to deploy and maintain, and less expensive than passive sampling programs. In the forest setting, semiochemical-baited flight intercept traps are a common active sampling tool.

In addition to their role in facilitating decision making in IPM programs, semiochemical-baited traps are also used for the direct control of pest populations (e.g., pheromone-based mating disruption [3]; mass trapping and attract-and-kill/infect [4]). They are also important components of eradication and containment programs designed to manage the establishment and/or spread of exotic species post-arrival [5]. Eradication and containment efforts are unlikely to succeed without affordable and effective sampling tools for several reasons. First, there is a negative correlation between the probability of successful containment and/or eradication and target population size. The more effective (i.e., sensitive) the sampling tools are, the earlier in the invasion process detection is likely to occur and the smaller population size is likely to be. Second, the iterative delineation of target population distribution is necessary, and third, without effective sampling tools, it is not possible to evaluate the success of containment and eradication efforts [5,6].

The factors known to influence the performance of semiochemical-baited intercept traps within program control include the number of insects attracted, proportion captured and retained and the target taxon distribution relative to the scale of sampling. Field trapping experiments have demonstrated that these factors can vary depending on the semiochemicals used to bait the trap [7,8,9,10] as well as with the trap type and design features [11,12,13]. The deployment protocol describes the temporal and spatial pattern of deploying the sampling technique. Primary among the spatial factors to consider in forest settings is trap location along environmental gradients, specifically horizontal edge–interior and vertical understory–canopy gradients. Although consistent patterns in gradient effects among forest Coleoptera have not yet emerged, it is clear that they have strong impacts on the diversity and abundance of the species captured [14,15,16,17,18,19,20,21,22,23,24,25,26,27,28,29,30].

The majority of studies that look at the impact of horizontal edge–interior and vertical understory–canopy gradients on the trap catches of forest beetles examine a single gradient (e.g., [29]; but see [30]). This study looked at the impact of both horizontal edge–interior and vertical understory–canopy gradients, and their interactions on the diversity of forest Coleoptera detected in traps. Specifically, we evaluated the effects of trap placement on the abundance and species richness of beetles commonly moved in solid wood packaging (Cerambycidae, Buprestidae, Curculionidae) [31,32] in two field experiments conducted in mixed coniferous broadleaf stands in New Brunswick, Canada. We ran experiment 2 to test the effect of forest edge on trap catches along a narrower forest opening than that at the site used in experiment 1 and also to make for a more robust study overall. We predicted that the species composition in traps would differ between the edge and interior traps as well as between the canopy and understory traps. Therefore, we also predicted that the total number of target taxa detected per number of traps deployed would be increased by using a diversity of trap placements (i.e., placing some traps along the stand edge and others in the stand interior, some traps in the canopy and others in the understory) rather than any single horizontal or vertical trap placement.

## 2. Materials and Methods

### 2.1. Field Trapping Experiments: General Background

We conducted experiment 1 at Keswick Ridge, NB (N 45.99618°, W66.87813°) from 16 May to 11 September 2014, and experiment 2 at Crabbe Mountain, Central Hainesville, NB (N46.12115°, W67.10524°) from 6 June to 6 September 2018. Common tree species at Keswick Ridge were *Acer saccharum* Marsh., *Fraxinus americana* L., *Populus tremuloides* Michx., and *Abies balsamea* (L.) Mill., with scattered *Pinus strobus* L., *Thuja occidentalis* L., and *Crataegus* spp. L. At Crabbe Mountain, common trees were *Betula alleghaniensis* Britton, *Fagus grandifolia* Ehrh., *A. saccharum*, *Acer rubrum* L., *Populus grandidentata* Michx., *A. balsamea*, and *Picea rubens* Sarg., with scattered *Tsuga canadensis* (L.) Carrière, and *P. strobus*.

In both experiments, we used Lindgren 12-funnel traps (Synergy Semiochemical Corporation, Delta, B.C) baited with a semiochemical lure combination designed to attract woodborers of broadleaved tree species: racemic 3-hydroxyhexan-2-one, racemic 3-hydroxyoctan-2-one, *syn*-2,3-hexanediols, *E,Z*-fuscumol, *E,Z*-fuscumol acetate, and ethanol [28]. Percentage purity, release rates and commercial sources of lures are in Table 1. The hydroxyketone and hexanediol lures were replaced after two months; the fuscumol, fuscumol acetate and ethanol lures were expected to last the entire season and were not replaced. All funnel traps were coated with Fluon (Bioquip, Ranch Domingo, CA, USA) (diluted by 50% in water) to reduce friction and increase beetle catches [33].

Traps in the understory were suspended from rope tied between two trees with the collecting cup 30–50 cm above the ground and the trap at least 1 m from the nearest tree. Canopy traps were suspended from rope thrown over a branch in the upper third of the tree canopy using a BigShot^®^ (SherillTree, Greensboro, NC, USA) following methods described in [34]. Trap-collecting cups contained a saturated solution of table salt in water to drown and preserve captured beetles, plus about 2 cm of solid salt in the bottom of the cup and a drop of liquid dish detergent to reduce surface tension. We checked and emptied the traps every 2–3 weeks and added water and/or salt to the collecting cups when required. Any traps found on the ground or otherwise damaged during trap checks were repaired and replaced; any catch in these traps was discarded but the specific trap number was noted so that data could subsequently be standardized per functional trapping day (see data analysis). Captured insects were stored at −10 °C until processed. Beetles in the families Cerambycidae, Buprestidae, Curculionidae and Dryophthoridae were determined to species whenever possible by using keys and illustrated guides [35,36] (http://www.barkbeetles.info/index.php) and voucher specimens were deposited in the Atlantic Forestry Centre insect collection.

### 2.2. Experiment 1—Effect of Horizontal and Vertical Gradients on Trap Catches

This was a modified factorial experiment testing the effects of both horizontal (forest edge vs. forest interior) and vertical (canopy vs. understory) trap placement on trap performance but with an additional open field trap placement. There were five treatments: (1) canopy–edge; (2) canopy–interior; (3) understory–edge; (4) understory–interior; and (5) open field. We replicated the treatments six times in randomized complete blocks with at least 30 m separating traps and blocks. Canopy traps ranged in height from 18 to 25 m. Interior traps were 30 m inside the forest edge that bordered a field. Open field traps were 30 m from the forest edge in a field and suspended from L-shaped steel rebar such that the traps were at the same height as understory traps, i.e., with collecting cups 30–50 cm above the ground. It was not feasible to add a sixth treatment of traps placed at a height of 18–25 m in the open field. The rebar poles were 2 m tall with a right angle bend that ran 0.4 m horizontally at the top and had a 0.3 m long bar welded at right angles about 30 cm from the bottom of the pole to make an inverted “t” shape which helped for pounding the poles into the ground.

### 2.3. Experiment 2—Effect of Horizontal Trap Position on Catches in Canopy Traps

There were three treatments: (1) interior; (2) edge; and (3) open. All traps were placed 12–15 m above the ground, either in the upper third of the tree canopy (interior and edge traps) or, for open traps, at about the same height in the center of a 14–15 m-wide open strip (old T-bar line) that cut through the forest running down the mountain. We suspended the open traps from the rope that was strung between the upper crowns of trees on opposite sides of the strip cut (using the BigShot^®^) so that the trap was 7–8 m from the forest edge on either side. Interior traps were placed 30 m inside the forest from the edge. We replicated each treatment 10 times in a randomized complete block design with 30 m spacing between blocks. Each block consisted of a linear transect of three traps that lay at a right angle to the old T-bar line opening that ran down the mountain. We used black funnel traps in five blocks and “EAB-green” funnel traps (Synergy Semiochemical Corporation, Delta, B.C.) in five blocks in an effort to increase species diversity in our traps [28]; trap colour was randomly assigned among blocks.

### 2.4. Data Analysis

For each trap, we pooled data across trap check dates to yield one total season catch per trap for each response variable. Response variables included the species richness and abundance of Buprestidae, Cerambycidae, Cerambycinae, Lamiinae, Lepturinae, Curculionidae, Scolytinae, non-Scolytine Curculionidae, and all target taxa combined, as well as the number of specimens per trap of individual species for which 15 or more specimens were captured. Due to traps occasionally being found on the ground during trap checks, data were missing for 14 days for three traps and 28 days for one trap at Keswick Ridge, and for 21 days for two traps at Crabbe Mountain. To correct bias for the traps that did not operate during the entire trapping period, we standardized data by dividing the total catch per trap by the number of days the trap was functional and multiplying this by the total number of days the experiment ran, e.g., 92 days for experiment 2 that ran from 6 June to 6 September.

Using these standardized data, we ran generalized linear mixed-effect models (PROC GLIMMIX) in SAS 9.4 for Windows (v. 6.2.9200, ©2002–2012, SAS Institute Inc., Cary, NC, USA) to test the effects of trap placement on each response variable, with trap placement considered fixed and blocks considered random. Blocks in which zero catch was recorded in all treatments were omitted from analysis. We ran models with Gaussian (normal), Poisson and negative binomial distributions and used the corrected Akaike information criterion (AICc) to select the model with the best fit. However, in 12 of 63 analyses, the model outputs using Poisson or negative binomial distributions were nonsense in spite of the AICc values suggesting they had the best fit, e.g., *F* values of zero or infinity; all 12 cases were with taxa with zero catches for all replicates in at least one trap position. For these cases, we instead reported the results from generalized linear models using the Gaussian distribution, after ensuring that residuals did not depart significantly from normality (Shapiro–Wilks test in SAS PROC GLM).

Data from experiment 1 were analyzed two ways: (1) simple 1-way ANOVA with five treatments replicated in blocks; and (2) as a 2 × 2 factorial crossing vertical position (canopy vs. understory) and horizontal position (edge vs. interior) with the open field trap treatment omitted. The factorial analysis was done to provide more statistical power and also to test for interactions between vertical and horizontal trap positions. For both experiments, EstimateS version 9.10 [37] was used to produce the sample-based (Coleman’s) rarefaction curves plotting the average number of species detected per number of traps deployed (Equation (5) in [38]) for each trap position. Violin plots were generated using ggplot2 in R [39].

## 3. Results

### 3.1. General Results

Altogether, we collected 142 different species of target taxa in traps (11 Buprestidae, 58 Cerambycidae, 72 Curculionidae, 1 Dryophthoridae) when both sites and experiments were pooled. Fifty-seven species were common to both sites (4 Buprestidae, 30 Cerambycidae, 23 Curculionidae), whereas 46 species were collected only at Keswick Ridge and 39 species were collected only at Crabbe Mountain. In experiment 1, at Keswick Ridge, we captured 103 species of target taxa (6 Buprestidae spp., 47 Cerambycidae spp., 49 Curculionidae spp., 1 Dryophthoridae) and 3747 individuals (51 Buprestidae, 693 Cerambycidae, 2956 Curculionidae, 47 Dryophthoridae) (Table S1, Supplementary Materials). In experiment 2, at Crabbe Mountain, we captured 96 species of target taxa (9 Buprestidae, 41 Cerambycidae, 45 Curculionidae, 1 Dryophthoridae) and 4458 individuals (117 Buprestidae, 1306 Cerambycidae, 3033 Curculionidae, 2 Dryophthoridae) (Table S2, Supplementary Materials). These totals and those in Tables S1 and S2 are raw count data, not standardized by number of days each trap was operating. All species were native to North America except for 12 species of Curculionidae (seven species of broad-nosed weevils in the subfamily Entiminae and five species of bark and ambrosia beetles in the subfamily Scolytinae) (Supplementary Tables S1 and S2). The two specimens of the clover root borer, *Hylastinus obscurus* (Marsham) (Scolytinae), collected at Crabbe Mountain, were the first records of its occurrence in New Brunswick [40]. There were 30 species at Keswick Ridge and 25 species at Crabbe Mountain represented by a single specimen, 26 and 29% of the total target species detected. Thirty-nine species were captured in high enough numbers to analyze by ANOVA and generalized linear models to determine the effects of trap position on mean catch (23 species at Crabbe Mountain, 27 species at Keswick Ridge, with 11 species common to both sites). Because we caught only one species of Dryophthoridae (*Dryophthorus americanus* Bedel) and it was formerly treated as a subfamily of Curculionidae, we pooled data on its catches with Curculionidae when estimating the species richness and abundance of Curculionidae in traps.

### 3.2. Experiment 1—Effect of Horizontal and Vertical Gradients on Trap Catches

**Figure 1:**
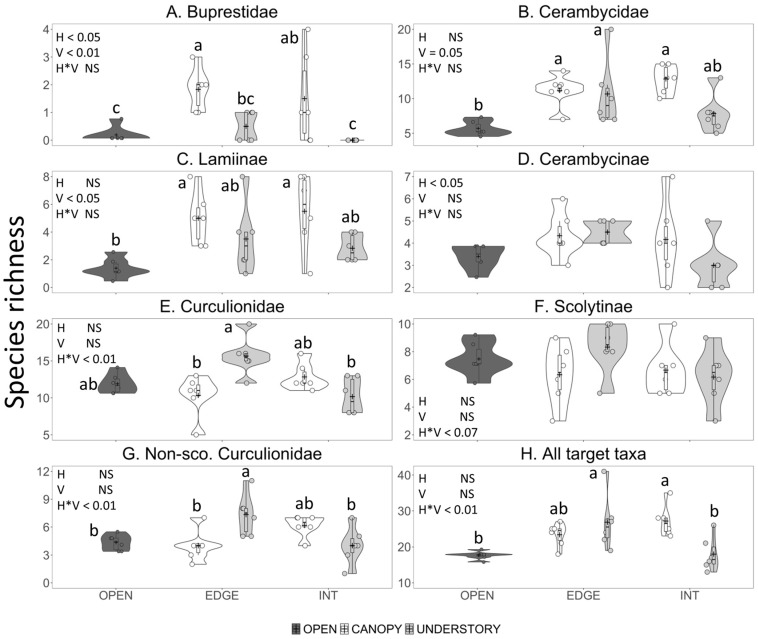
Violin plots showing the effect of horizontal (open field vs. forest edge vs. interior) and vertical (canopy vs. understory) trap placement on species richness per trap of: (**A**) Buprestidae; (**B**) Cerambycidae; (**C**) Lamiinae; (**D**) Cerambycinae; (**E**) Curculionidae; (**F**) Scolytinae; (**G**) Non-scolytine Curculionidae; and (**H**) All target taxa combined. Count data are shown by the jittered dots, the interquartile range and median are shown by the vertical bar and black dash, respectively, and the mean is shown by the plus sign. Traps were black, Fluon-treated 12-funnel Lindgren traps set in a mixed broadleaf–coniferous forest near Keswick Ridge, New Brunswick, 2014. Significance of horizontal (H) or vertical (V) trap position and their interaction (H × V) were determined by generalized linear models with the open field treatment omitted. Means with different letters differed significantly (Tukey–Kramer multiple comparisons of least square means from single factor generalized linear models with all five trap position treatments).

**Figure 2:**
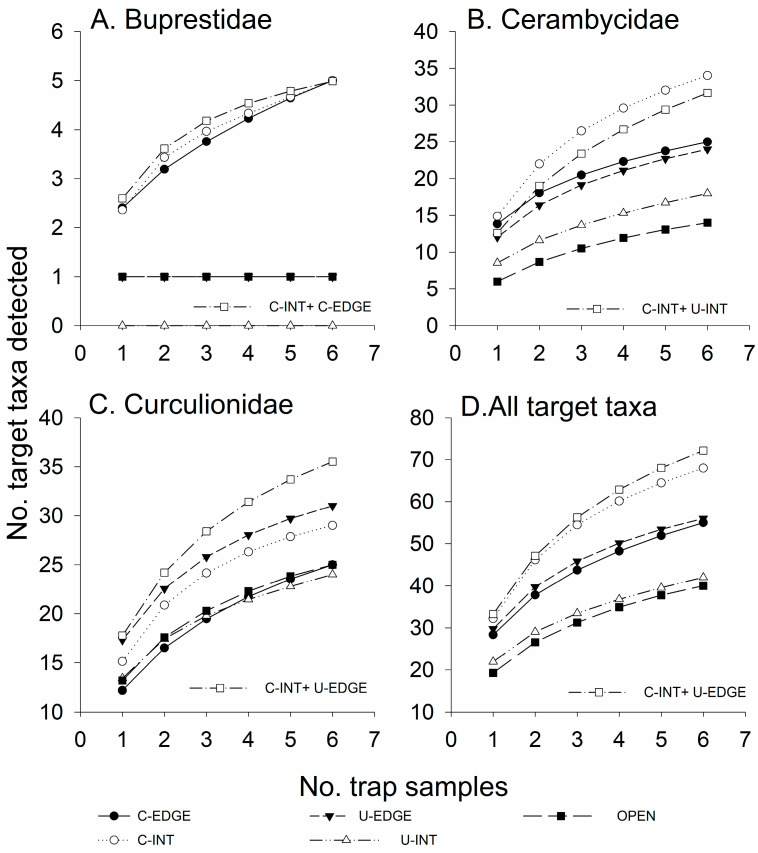
Mean number of species detected per number of trap samples (Coleman’s rarefaction) in black Fluon-treated 12-funnel Lindgren traps placed in the upper canopy along the forest edge (C–EDGE) or 30 m inside the forest (C–INT), or in the understory along the forest edge (U–EDGE) or 30 m inside the forest (U–INT), or in an open field 30 m from the forest edge (OPEN): (**A**) Buprestidae, (**B**) Cerambycidae, (**C**) Curculionidae, and (**D**) All target taxa combined. The two-treatment combination that detected the most species per sampling effort is depicted by the open square symbols.

**Figure 3:**
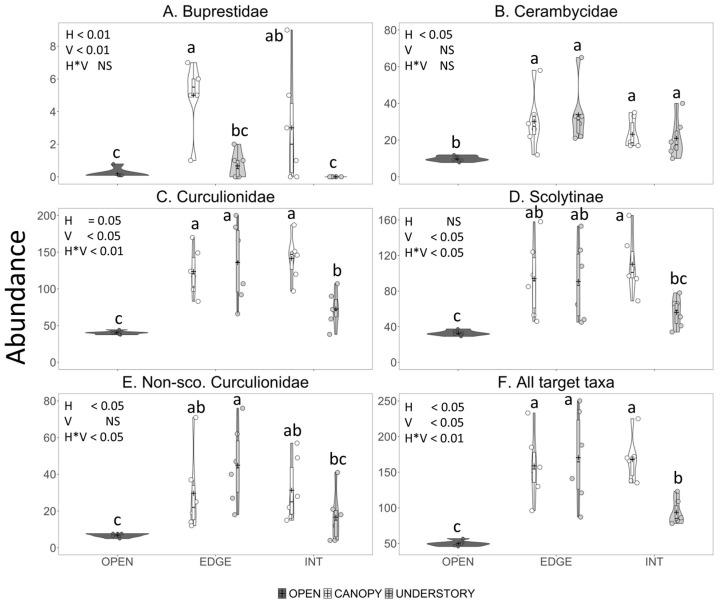
Violin plots showing the effect of horizontal (open field vs. forest edge vs. interior) and vertical (canopy vs. understory) trap placement on the abundance per trap of: (**A**) Buprestidae; (**B**) Cerambycidae; (**C**) Curculionidae; (**D**) Scolytinae; (**E**) Non-scolytine Curculionidae; and (**F**) All target taxa combined. Count data are shown by the jittered dots, the interquartile range and median are shown by the vertical bar and black dash, respectively, and the mean is shown by the plus sign. Traps were black, Fluon-treated 12-funnel Lindgren traps set in a mixed broadleaf–coniferous forest near Keswick Ridge, New Brunswick, 2014. Significance of horizontal (H) or vertical (V) trap position and their interaction (H × V) were determined by generalized linear models with the open field treatment omitted. Means with different letters differed significantly (Tukey–Kramer multiple comparisons of least square means from single factor generalized linear models with all five trap position treatments).

Species richness and abundance of bark- and woodboring beetles captured in traps were significantly affected by horizontal and vertical trap position and their interaction; trap positions that performed the best differed among taxa (Figure 1, Figure 2 and Figure 3; Table 2). According to the AICc values, data on species richness were fit best with the Poisson distribution in five cases and the Gaussian distribution in four cases, whereas the data on abundance were fit best with the Poisson distribution in 15 cases, the negative binomial distribution in 19 cases and the Gaussian distribution in two cases.

#### 3.2.1. Species Richness

Both horizontal and vertical trap placement significantly affected the number of buprestid species captured in the traps, with greater mean species richness in the traps in the canopy and along the forest edge than in the understory or inside the forest (Figure 1A, Table 2). Coleman’s rarefaction curves show a similar trend in the mean number of species detected per number of traps for each trap position (Figure 2A). Vertical trap placement had the most obvious effect, with canopy traps detecting five buprestid species per six traps, both inside the forest and on the edge, compared to only one species, *Dicerca divaricata* (Say), detected in six traps in the understory and open field traps (Table 3). No buprestids were detected in understory traps placed inside the forest.

The factorial analysis showed that the cerambycid species richness was significantly affected by vertical but not horizontal trap placement: on average, more species were collected in black 12-funnel traps in the canopy than in the understory (Figure 1B; Table 2). However, when the open field treatment was included and the data were analyzed by one-way ANOVA followed by the Tukey–Kramer multiple comparison test, mean cerambycid richness did not differ significantly among any trap position in the forest but was significantly greater in the edge–canopy-, edge–understory-, and interior–canopy-traps than in the open field traps (Figure 1B).

Effect of trap position on the detection of cerambycid species is more clearly seen in the species’ accumulation curves (Figure 2B). In a total of six traps per trap position, traps placed in the field or in the understory inside the forest detected the fewest species (14 and 18, respectively), traps along the forest edge detected 24–25 species regardless of vertical trap placement, and the canopy traps inside the forest detected 34 species (Figure 2B).

There were differences among cerambycid subfamilies in the effects of trap placement on species richness. The factorial analysis showed that vertical trap position significantly affected the species richness of Lamiinae (canopy > understory) (Figure 1C) but not Cerambycinae (Figure 1D), whereas the horizontal position significantly affected the species richness of Cerambycinae (edge > interior) but not Lamiinae (Table 2). However, in the less powerful one-way ANOVAs, the only significant differences in Lamiinae species richness were between the canopy traps and the open field treatment (Figure 1C), and Cerambycinae species richness did not differ among any trap position (Figure 1D). Neither vertical nor horizontal trap position affected the species richness of Lepturinae (Table 2), and too few species of Spondylidinae (three spp.) were captured to warrant analysis.

Species richness of Curculionidae was significantly affected by the interaction between horizontal and vertical trap placement: understory traps detected more species than canopy traps when placed along the forest edge but not when placed inside the forest (Figure 1E, Table 2). Trends were similar for Scolytinae (Figure 1F) and non-scolytine Curculionidae (Figure 1G). The species’ accumulation curves showing the most effective trap positions for detecting Curculionidae were understory traps on the forest edge followed by canopy traps inside the forest (Figure 2C). However, a combination of understory–edge and canopy–interior traps (Figure 2C, open squares) detected more species of Curculionidae than did any single trap position.

Species richness of total target taxa was significantly affected by the interaction between horizontal and vertical trap placement (Table 2). Canopy traps inside the forest and understory traps on the forest edge detected more species of target taxa than did traps in the understory inside the forest or in the open field (Figure 1H). The species’ accumulation curves show that the rate of species detection per sampling effort was greater for canopy traps inside the forest than for any other single trap position, with 68 species detected in six traps, compared to 55–56 species detected in six traps on the forest edge in either the canopy or understory, and 40–42 species detected in six traps in the understory inside the forest or in the open field (Figure 2D). As we predicted, placing traps in more than one trap position increased the total number of species detected. The combination of three canopy traps inside the forest and three understory traps on the forest edge detected more species of target taxa (72) than any single trap position (Figure 2D).

#### 3.2.2. Abundance

Mean catch of buprestid specimens was significantly affected by horizontal and vertical trap position with the greatest catch in canopy traps on the forest edge and very low catches in understory traps inside the forest, at the forest edge and in the open field (Figure 3A; Table 2). Mean catch of *D. divaricata,* the only buprestid species caught in sufficient numbers to warrant analysis, followed a similar trend, with significantly lower catches in understory–interior traps than in any other trap position (Table 3).

Abundance of cerambycids in traps was significantly affected only by horizontal trap position, with greater catches in traps along the forest edge than inside the forest; traps in the open field captured even fewer cerambycids (Figure 3B, Table 2). Trap position did not affect the abundance of Lamiinae, Cerambycinae, or Lepturinae in traps (Table 2), but did significantly affect the mean catch of ten cerambycid species (Table 3). Abundance of *Clytus marginicollis* Laporte and Gory, *Microclytus compressicollis* (Castelneau and Gory), *Neoclytus a. acuminatus* (Fabricius) (Cerambycinae) and *Aegomorphus modestus* (Gyllenhal) (Lamiinae) was affected by vertical trap position, with greater mean catches in canopy traps than understory traps for all but *M. compressicollis* (Table 3). Mean catch of *Astylopsis macula* (Say) (Lamiinae) was significantly greater in the traps placed along the forest edge than inside the forest (Table 3). Mean catch of *Cyrtophorus verrucosus* (Cerambycinae) was affected by both the vertical and horizontal trap position, with greater abundance in traps on the forest edge vs. inside the forest, and in the canopy vs. the understory (Table 3). The catch of two species was affected by vertical and horizontal trap position and their interaction: most specimens of *Pogonocherus pencillatus* LeConte (Lamiinae) were captured in canopy traps on the forest edge whereas most specimens of *Tetropium cinnamopterum* Kirby (Spondylidinae) were captured in understory traps inside the forest (Table 3).

Abundance of Curculionidae was significantly affected by horizontal and vertical trap position and their interaction (Table 2), with high catches in both canopy and understory traps on the forest edge and in canopy traps inside the forest, significantly lower catches in understory traps inside the forest, and even lower catches in the field traps (Figure 3C). The interaction between the horizontal and vertical position was also significant for the abundance of Scolytinae and non-scolytine Curculionidae (Table 2). Mean catch of scolytines was significantly greater in the canopy traps than understory traps when the traps were placed inside the forest but not when placed along the forest edge (Figure 3D), whereas non-scolytine curculionids were significantly more abundant on the forest edge than inside the forest in understory traps but not in canopy traps (Figure 3E).

Trap position significantly affected mean catch of ten species of Curculionidae (four non-scolytine Curculionidae and six Scolytinae) (Table 3). Vertical trap position affected more species than did horizontal trap position. Mean catches of *Phyllobius oblongus* (L.) (Entiminae), *Orthotomicus caelatus* (Eichhoff)*, Xyloterinus politus* (Say), and *Anisandrus obesus* LeConte (Scolytinae) were significantly greater in the understory than in the canopy, whereas mean catches of *Hylesinus aculeatus* (Say) and *Polygraphus rufipennis* (Kirby) (Scolytinae) were significantly greater in the canopy than the understory (Table 3). Mean catch of *H. aculeatus* was also significantly affected by horizontal trap position with greater catches on the forest edge than inside the forest (Table 3). The interaction between horizontal and vertical trap position significantly affected catch of three species. Mean catches of *Polydrusus formosus* (Meyer) (Entiminae) and *Anisandrus sayi* (Hopkins) (Scolytinae) in canopy traps were double those in understory traps when traps were placed inside the forest but were nearly the same in canopy and understory traps along the forest edge (Table 3). Conversely, the mean catch of *Dryophthorus americanus* (Bedel) (Dryophthoridae: Rhynchophorinae) was greater in the canopy than the understory traps, but only for traps on the forest edge and not inside the forest (Table 3). Mean catch of *Stenoscelis brevis* (Boheman) (Cossoninae) was significantly lower in the open field traps than in any other trap position (Table 3).

Abundance of the total target taxa was significantly affected by horizontal and vertical trap position and their interaction (Table 2) with relatively high abundance in traps on the forest edge regardless of trap height, as well as in canopy traps inside the forest, significantly lower catches in understory traps inside the forest, and lowest catches in field traps (Figure 3F).

### 3.3. Experiment 2—Effect of Horizontal Trap Position on Catches in Canopy Traps

**Figure 4:**
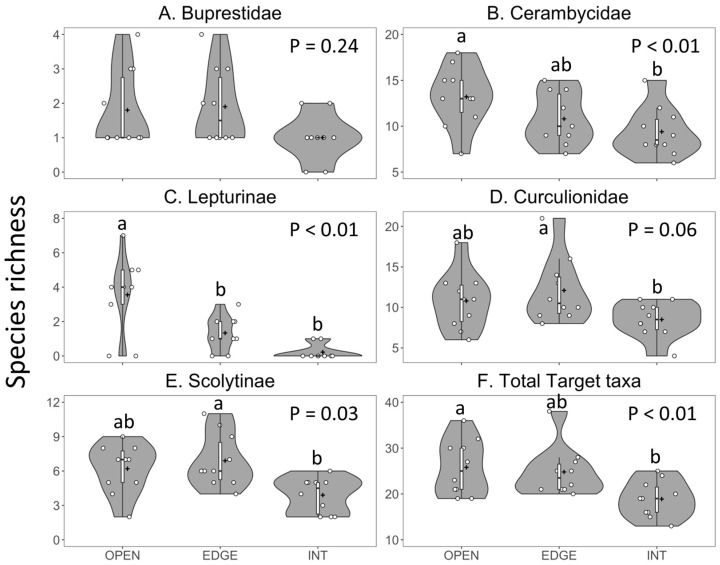
Violin plots showing the effect of horizontal trap position (forest opening vs. forest edge vs. forest interior) on species richness per trap of: (**A**) Buprestidae; (**B**) Cerambycidae; (**C**) Lepturinae; (**D**) Curculionidae; (**E**) Scolytinae; and (**F**) all target taxa. Count data are shown by the jittered dots, the interquartile range and median are shown by the white vertical bar and black dash, respectively, and the mean is shown by the plus sign. All the Fluon-treated 12-funnel Lindgren traps were placed in the upper tree canopy or at the equivalent height in a 14–15 m-wide opening that ran the length (top to bottom) of a mixed broadleaf–coniferous forest on Crabbe Mountain, New Brunswick, 2018. Equal numbers of green and black traps were used. Means with different letters differed significantly (Tukey–Kramer multiple comparisons of least square means from single factor generalized linear models, *p* < 0.05).

**Figure 5:**
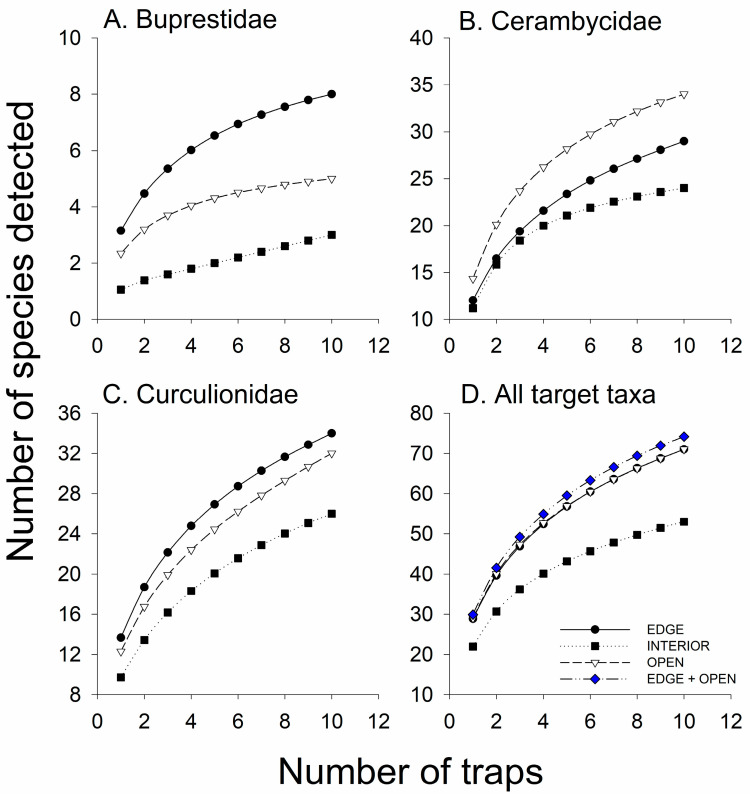
Mean number of species of: (**A**) Buprestidae, (**B**) Cerambycidae, (**C**) Curculionidae, and (**D**) all target taxa detected per number of trap samples (Coleman’s rarefaction) in Fluon-treated 12-funnel Lindgren traps placed along the forest edge (EDGE) vs. 30 m inside the forest (INT) vs. the center of a 14–15 m-wide opening (OPEN) running the length (top to bottom) of a mixed broadleaf–coniferous forest on Crabbe Mountain, New Brunswick, 2018. All traps were in the upper tree canopy or at the equivalent height in the opening. Equal numbers of green and black traps were used. Note: the rarefaction curves for all target taxa (D) were almost identical for EDGE and OPEN traps, resulting in overlapping curves and obscuring of the OPEN symbols.

**Figure 6:**
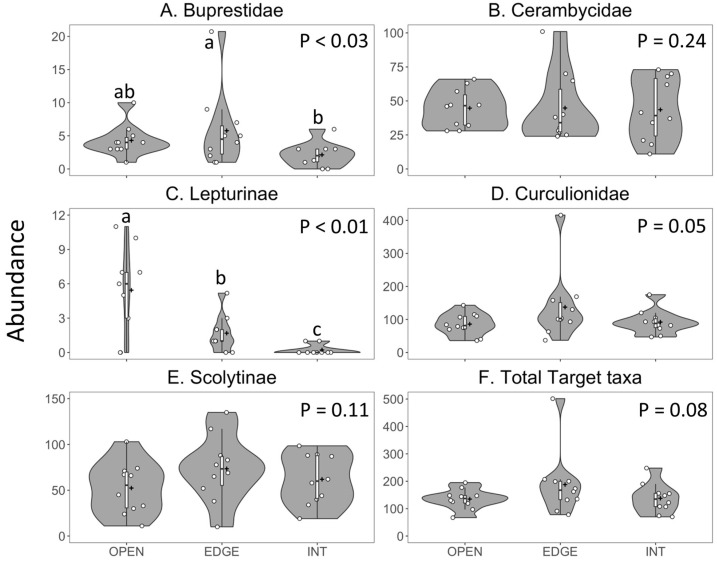
Violin plots showing the effect of horizontal trap position (forest opening vs. forest edge vs. forest interior) on the abundance per trap of: (**A**) Buprestidae; (**B**) Cerambycidae; (**C**) Lepturinae; (**D**) Curculionidae; (**E**) non-scolytine Curculionidae; and (**F**) total target taxa. Count data are shown by the jittered dots, the interquartile range and median are shown by the white vertical bar and black dash, respectively, and the mean is shown by the plus sign. All the Fluon-treated 12-funnel Lindgren traps were placed in the upper tree canopy or at the equivalent height in a 14–15 m-wide opening that ran the length (top to bottom) of a mixed broadleaf–coniferous forest on Crabbe Mountain, New Brunswick, 2018. Equal numbers of green and black traps were used. Means with different letters differed significantly (Tukey–Kramer multiple comparisons of least square means from single factor generalized linear models, *p* < 0.05).

Horizontal position of upper canopy traps significantly affected the species richness (Table 4, Figure 4 and Figure 5) and abundance (Table 4, Figure 6) of target taxa captured, but the effects differed among beetle families. Data on the species richness were fit best with the Poisson distribution in seven cases, the Gaussian distribution on log-transformed data in one case, and the Gaussian distribution on raw data in one case, whereas the data on abundance were fit best with the negative binomial distribution in 17 cases, the Poisson distribution in 13 cases and the Gaussian distribution in two cases.

#### 3.3.1. Species Richness

The mean number of buprestid species per trap did not differ among horizontal trap position (Figure 4A). However, the rarefaction curves clearly show that the traps placed along the forest edge detected more buprestid species per number of trap samples than did the traps in the forest opening or interior (Figure 5A).

Cerambycid species richness was significantly greater in the open traps than in the traps inside the forest, with intermediate richness in the traps along the forest edge (Table 4, Figure 4B and Figure 5B). Horizontal trap position did not significantly affect the species richness within the subfamilies Cerambycinae and Lamiinae (Table 4) but the species richness of Lepturinae was significantly greater in open traps than in traps on the edge or inside the forest (Table 4, Figure 4C).

Curculionid species richness was significantly greater in traps placed on the forest edge than in its interior, whereas the species richness in open traps did not differ significantly from that in the edge or interior traps (Table 4, Figure 4D and Figure 5C). The trend was the same for Scolytinae (Table 4, Figure 4E) but there was no effect of horizontal trap position on the species richness of non-scolytine Curculionidae (Table 4).

Species richness of total target taxa was significantly greater in the traps placed in the open than in the traps placed inside the forest and the traps at the forest edge were intermediate (Table 4, Figure 4F). The rarefaction curves for the traps in the opening and on the forest edge were nearly identical, accumulating the same numbers of target taxa per trap sample (Figure 5D, curves overlap, obscuring the symbols for OPEN traps). Using a combination of traps on the forest edge as well as in the opening detected slightly more species (Figure 5D, diamond symbols). However, within each beetle family, using a combination of two trap positions, e.g., five edge traps and five interior traps, did not increase the total number of species detected (data not shown). Thus, the results of experiment 2 did not support our prediction that placing traps in more than one trap position would increase the number of target taxa detected.

#### 3.3.2. Abundance

Abundance of buprestids was significantly greater in the traps on the forest edge than in its interior, with intermediate catches in open traps (Table 4, Figure 6A). Mean catch per trap of *D. divaricata* was unaffected by horizontal trap position (Table 5).

Mean abundance of Cerambycidae (Figure 6B), Cerambycinae and Lamiinae were not affected by horizontal trap position (Table 4), but the abundance of Lepturinae was significantly greater in open traps than in edge or interior traps, and greater in edge traps than in interior traps (Table 4, Figure 6C). Trap position significantly affected the mean catch per trap of *Glycobius speciosus* (Say) (Cerambycinae) and *A. macula,* with near significant (*p* = 0.07) effects on the catch of *Anthophylax cyaneus* (Haldeman) (Lepturinae); all three species were captured in greater numbers in traps placed in the forest opening than in the forest interior (Table 5).

Abundance of Curculionidae in traps was significantly affected by horizontal trap position with greatest catches on the forest edge, but the means were not separated (Table 4, Figure 6D). The trends were similar but not significant for both Scolytinae (Figure 6E) and non-scolytine Curculionidae (data not shown) (Table 4). Trap position significantly affected the mean catch of five Curculionidae species (Table 5). Mean catches of *Phyllobius oblongus* (L.) (Entiminae) and *Xyleborinus attenuatus* (Blandford) (Scolytinae) were significantly greater in open traps than in traps on the edge or interior of the forest whereas mean catches of *Crypturgus borealis* Swaine and *Pseudopityophthorus minutissimus* (Zimmerman) (Scolytinae) were significantly greater in traps on the forest edge than in traps in the forest opening or interior (Table 5). The latter trend was also true for the mean catch of *Polydrusus cervinus* (L.) (Entiminae) but the means were not separated (Table 5).

Mean abundance of the total target taxa was greatest in the traps along the forest edge but the effect of trap position was only marginally significant (*p* = 0.08) (Table 4; Figure 6F).

## 4. Discussion

IPM programs incorporate protocols to guide management decisions and these protocols involve the assessment of pest population density. As a result, the development and optimization of cost-effective sampling programs is a critical step in the management of insect pests. Ideally, this process involves both the development of the sampling technique and the deployment protocol to inform the use of the sampling technique. While semiochemical-baited flight intercept traps have been developed for many forest insect pests, deployment protocols have received far less attention. This study characterized the impact of deployment protocol, specifically trap placement along horizontal edge–interior and vertical understory–canopy gradients, on the capture of forest Coleoptera from the families of Buprestidae, Cerambycidae and Curculionidae. Both gradients had significant effects on the diversity and abundance of all three families; however, the pattern of gradient effects varied between experiments.

Edge and interior traps associated with large forest openings (>100 m-wide, experiment #1) captured a higher species richness of Buprestidae than the traps in the adjacent open field while there was no effect of horizontal gradients associated with smaller forest openings (ca. 15 m, experiment #2). The pattern of horizontal gradient effects on the abundance of Buprestidae were similar for transects of the large and small forest openings.

For the Cerambycidae, the interior and edge traps associated with large forest openings captured a higher species richness and abundance of Cerambycidae than the traps in the adjacent open field. Conversely, with small forest openings, traps in the opening captured a higher species richness than the traps in the forest interior (edge traps were intermediate) and there was no effect of horizontal gradients on Cerambycidae abundance.

In general, the pattern of Curculionidae species richness and abundance was the same for the horizontal gradients associated with large and small forest openings. The effects of horizontal gradients in large forest openings in this study are generally consistent with the available literature [14,16,18,21,41,42]. Although a meta-analysis of the effects of forest edges on insect herbivores observed that both the abundance and species richness were higher at the forest edges [43], heterogeneity in the effects of horizontal edge gradients exists. Both bark beetle and weevil abundance have been reported to be higher in the forest interior [44,45] and higher abundance of Buprestidae in forest openings than at forest edges have been reported [17]. Allison et al. [29] reported three different patterns of horizontal edge interior gradient effects for species of Cerambycidae. Two species were more abundant in open fields than the forest interior, one species more abundant at the forest edge than the interior and five species more abundant in the forest interior than in the adjacent open field.

In almost all cases, the species’ accumulation curves did not reach an asymptote in either experiment, indicating that 6–10 traps were not sufficient to detect all target species present at the sites, even when traps were deployed at more than one position. This is an important consideration for regulatory agencies when planning surveillance protocols for exotic bark- and woodborers, but with limited budgets, increasing the number of traps per site may come at the cost of reducing the number of sites surveyed per year.

The two experiments looking at gradient effects in this study were conducted in separate years and locations, 4 years and approximately 24 km apart. One hypothesis is that the different gradient effects are a result of different communities of species from the three families being sampled. There was considerable overlap in the species sampled in the two experiments. For example, four of the 11 species of Buprestidae, 30 of the 58 species of Cerambycidae and 23 of the 71 species of Curculionidae captured were common to both experiments. Of the species captured in high enough numbers in both experiments for analysis, different patterns of horizontal gradient effects were observed in six of 11 species (Table 3 and Table 5). In terms of abundance, the Buprestidae species that were captured in both experiments made up 66% and 63% of the total buprestid catch in Experiments 1 and 2, respectively. The species of Cerambycidae and Curculionidae captured in both experiments made up 71% and 80% and 94% and 73% of the total catch per family in Experiments 1 and 2, respectively. Cumulatively, these results suggest that the different patterns of horizontal gradient effects observed in the two experiments are not due to differences in the community of beetles sampled.

Insect responses to vertical and horizontal forest gradients are difficult to predict due to variation among species in resource requirements that are poorly understood. Additionally, insect resource requirements can also be characterized by rapid temporal and fine scale variability. Although gradient effects have been studied in forests with different edge characteristics and variable surrounding matrices, there is no consensus among studies. Not surprisingly, the mechanisms responsible for gradient effects are poorly understood. Murcia [46] recognized three types of edge effects experienced by habitats adjacent to edges: abiotic effects, direct biotic effects and indirect biotic effects. The forest edges from this study and in most managed forests, represent an abrupt transition between two relatively homogeneous habitats (horizontal—open field and forest; vertical—understory and canopy) although the horizontal transition is typically more abrupt. Edge effects are due to abiotic and biotic (direct and indirect) effects originating in one habitat that influence the adjacent habitat. The portion of the habitat with an observable edge effect is the edge zone. The size of forest edge zones can vary with the size of the adjacent non-forest habitat, and this likely explains the different patterns of gradient effects observed in the large and small forest openings in this study. For example, Ulyshen et al. [16] compared the species richness and abundance of several groups of forest Coleoptera in forest gaps of three sizes (0.13 ha, 0.26 ha and 0.5 ha) and observed an effect of gap size on species richness (0.13 ha gaps <0.26 and 0.5 ha gaps). Although that study does not indicate gap dimensions, if we assume the gaps were square they would have been ca. 36 × 36 m, 51 × 51 m and 71 × 71 m suggesting that the gap size effects of that study and ours occur over similar spatial scales.

This study also observed vertical understory–canopy gradient effects. In general, the abundance and species richness of Buprestidae were higher in the canopy than in the understory. No effect of trap height on the abundance of Cerambycidae was observed. The effect of trap height on the species richness of Cerambycidae and on both the abundance and species richness of Curculionidae varied with trap location along the horizontal gradient. Cerambycidae species richness and Curculionidae abundance were greater in canopy than understory traps in the forest interior but there were no effects on either variable at the forest edge. Conversely, Curculionidae species richness was higher in canopy than understory traps at the forest edge while there was no effect of trap height on species richness in the forest interior and abundance was higher in canopy than understory traps in the forest interior. In temperate forests, variable canopy–understory gradient effects have been reported [17,22,23,26,28,47,48,49,50,51,52,53]. As observed in this study, context-dependent vertical gradient effects have been reported. For example, Vodka and Cizek [21] observed that the diversity of saproxylic species was higher in the understory than in the canopy at the forest edge while the opposite pattern was observed in the forest interior (although not statistically significant).

## 5. Conclusions

In terms of frequency and impact, bark and woodboring beetles are among the most significant insect threats to forest health globally [54,55]. Efforts to characterize changes in forest insect species ranges and population density are increasing due to growing concern over how species will respond to climate change [56], how to best maintain biodiversity [57] and the spread of alien species [58]. The development of effective survey and detection tools is a critical step in these efforts, but ideally, is also followed by studies describing the patterns of gradient effects (both vertical canopy–understory and horizontal edge–interior gradients). This study demonstrates strong gradient effects for three important families of bark and woodboring beetles (Curculionidae, Buprestidae and Cerambycidae), that these gradient effects vary among and within these families and that gradient effects can be context dependent. The results of this study clearly indicate that survey and detection efforts for species from these families must consider trap placement along horizontal and vertical gradients. Ideally, monitoring programs for bark and woodboring beetles should deploy traps at multiple locations along both vertical understory–canopy and horizontal edge–interior gradients.

## Figures and Tables

**Table 1:** List of the semiochemical lures used with percentage purity, release rates, and sources.

Semiochemical	Release Device	Purity (%)	Release Rate (mg/d) at 20 °C	Source ^3^
Racemic 3-hydroxyhexan-2-one	Pouch	99% ^1^	20–25	Bedoukian Research Danbury, CT/Contech Enterprises (Scott’s Canada), Delta, BC
Racemic 3-hydroxyoctan-2-one	Pouch	99% ^1^	20–25	Bedoukian Research/Contech Enterprises (Scott’s Canada)
Racemic *syn*-2,3-hexanediols	Pouch	99% ^1^	1–2	Atlantic Forestry Centre, Fredericton, NB/Contech Enterprises (Scott’s Canada)
(*E/Z*)-fuscumol	Rubber septa	99% ^2^	0.5–2	Sylvar Technologies, Fredericton, NB
(*E/Z*)-fuscumol acetate	Rubber septa	99% ^2^	0.5–2	Sylvar Technologies
Ethanol ultra high release rate (UHR) lure	Pouch	99% ^2^	300–400	Contech Enterprises (Scott’s Canada)

^1^ Determined at Canadian Forest Service, Atlantic Forest Centre, Fredericton, NB. ^2^ Supplied by manufacturer. ^3^ Both hydroxyketones were synthesized by Bedoukian Research and the *syn*-2,3-hexanediols were synthesized by Peter Mayo, Atlantic Forestry Centre; all three compounds were loaded into release devices by Contech Enterprises/Scott’s Canada.

**Table 2:** Results of generalized linear models testing the effects of horizontal and vertical trap position on species richness and the abundance of Buprestidae, Cerambycidae, and Curculionidae, and sub-taxa, collected in black 12-funnel Lindgren traps in a mixed broadleaf–coniferous forest near Keswick Ridge, NB, Canada in 2014.

Figure	Variable	Horizontal		Vertical		Hor x Vert		Model
		*F*_1, 20_	*p*	*F*_1, 20_	*p*	*F*_1, 20_	*p*	
**Buprestidae**	Richness	6.8	0.02	33.7	0.00	0.1	0.79	Gaussian, log
	Abundance	10.3	0.01	46.3	0.00	0.6	0.45	Gaussian, log
**Cerambycidae**	Richness	0.4	0.53	4.5	0.05	3.1	0.09	Poisson
	Abundance	5.1	0.04	0.0	0.99	0.4	0.53	Neg. binomial
Lamiinae	Richness	0.1	0.78	6.1	0.02	0.6	0.47	Poisson
	Abundance	2.7	0.12	2.6	0.12	0.5	0.51	Neg. binomial
Cerambycinae	Richness	5.3	0.03	1.2	0.28	2.5	0.13	Gaussian, log
	Abundance	3.9	0.06	1.5	0.24	0.1	0.81	Neg. binomial
Lepturinae	Richness	0.3	0.57	0.3	0.57	2.8	0.11	Poisson
	Abundance	1.9	0.19	0.0	0.97	1.9	0.19	Neg. binomial
**Curculionidae**	Richness	2.0	0.17	1.9	0.18	23.8	0.00	Gaussian, log
	Abundance	4.2	0.05	5.8	0.03	10.2	0.01	Neg. binomial
Scolytinae	Richness	2.0	0.17	1.3	0.26	3.7	0.07	Gaussian
	Abundance	1.2	0.29	5.5	0.03	4.5	0.05	Neg. binomial
Non-Scolytine	Richness	0.7	0.40	0.7	0.40	16.5	0.00	Gaussian
Curculionidae	Abundance	5.2	0.03	0.1	0.81	7.2	0.01	Neg. binomial
**All target taxa**	Richness	2.1	0.16	2.6	0.12	10.6	0.00	Poisson
	Abundance	6.7	0.02	5.9	0.03	9.6	0.01	Neg. binomial

**Table 3:** Mean catch per trap of target taxa in black 12-funnel Lindgren traps placed in the upper canopy or understory, on the forest edge or in its interior, or in an adjacent open field, at Keswick Ridge, NB, from 16 May to 11 September 2014. Species collected in insufficient numbers for analysis by generalized linear models are not shown. Values of *F*, degrees of freedom and *p* are from factorial models that excluded the open field trap treatment. Means followed by different letters were significantly different, Tukey–Kramer’s multiple comparison test on least square means from 1-way ANOVA models included the open field trap treatment (experiment-wise error = 0.05.).

Family	Species	Canopy		Understory				Horizontal		Vertical		Hor*Vert	
		Edge	Interior	Edge	Interior	Open	df	*F*	*p*	*F*	*p*	*F*	*p*
Buprestidae	*Dicerca divaricata*	3.17 ± 0.87 a	0.83 ± 0.48 a	0.67 ± 0.33 a	0 ± 0 b	0.17 ± 0.17 a	1,20	10.90	<0.01	12.70	<0.01	1.05	0.32
Cerambycidae	*Clytus marginicollis*	1.50 ± 0.56	1.33 ± 0.33	0.33 ± 0.21	0 ± 0	1.50 ± 0.62	1,20	0.64	0.43	15.90	<0.01	0.07	0.79
Cerambycidae	*Clytus ruricola*	1.83 ± 0.70	1.00 ± 0.52	2.50 ± 0.62	1.17 ± 0.31	0.33 ± 0.21	1,20	4.01	0.06	0.46	0.50	0.05	0.82
Cerambycidae	*Cyrtophorus verrucosus*	4.33 ± 1.58 a	1.00 ± 0.37 b	1.67 ± 0.88 ab	0.17 ± 0.17 b	1.67 ± 0.49 ab	1,20	10.88	<0.01	5.78	0.03	0.54	0.47
Cerambycidae	*Microclytus compressicollis*	0.67 ± 0.49 ab	0.83 ± 0.48 ab	1.33 ± 0.42 ab	2.67 ± 0.88 a	0.00 b	1,20	1.32	0.26	5.41	0.03	0.35	0.56
Cerambycidae	*Neoclytus acuminatus*	2.00 ± 0.58	1.75 ± 1.44	0 ± 0	0 ± 0	0.25 ± 0.25	1,12	0.77	0.40	15.37	<0.01	0.77	0.40
Cerambycidae	*Phymatodes maculicollis*	3.50 ± 2.74	2.67 ± 0.99	10.17 ± 1.92	7.17 ± 2.80	2.83 ± 1.17	1,20	0.44	0.52	3.04	0.10	0.01	0.92
Cerambycidae	*Aegomorphus modestus*	1.80 ± 0.91	1.00 ± 0.55	0 ± 0	0 ± 0	0.6 ± 0.24	1,16	0.77	0.39	9.42	0.01	0.77	0.39
Cerambycidae	*Astylopsis macula*	3.17 ± 1.11	1.50 ± 0.56	7.33 ± 4.65	1.00 ± 0.52	1.17 ± 0.48	1,20	7.69	0.01	0.19	0.67	1.59	0.22
Cerambycidae	*Monochamus scutellatus*	1.17 ± 0.54	0.83 ± 0.40	1.50 ± 1.02	0 ± 0	0 ± 0	1,20	2.68	0.12	0.20	0.66	1.08	0.31
Cerambycidae	*Pogonocherus pencillatus*	4.00 ± 1.58 a	0.60 ± 0.24 b	0.20 ± 0.20 b	0 ± 0 b	0 ± 0 b	1,16	13.00	<0.01	28.40	<0.01	7.53	0.01
Cerambycidae	*Urgleptes signatus*	2.17 ± 1.08	2.67 ± 1.02	2.17 ± 0.65	3.17 ± 0.83	0.17 ± 0.17	1,20	1.28	0.27	0.11	0.74	0.11	0.74
Cerambycidae	*Centrodera decolorata*	1.33 ± 1.15	0.83 ± 0.31	1.33 ± 0.61	1.17 ± 0.60	0 ± 0	1,20	0.31	0.59	0.10	0.76	0.10	0.76
Cerambycidae	*Tetropium cinnamopterum*	0 ± 0	0 ± 0	0.67 ± 0.33	3.67 ± 0.88	0.5 ± 0.50	1,8	19.20	<0.01	40.03	<0.01	19.2	<0.01
Curculionidae	*Stenoscelis brevis*	4.17 ± 1.30 a	2.00 ± 0.51 a	4.17 ± 1.45 a	3.33 ± 1.02 a	0 ± 0 b	1,20	2.46	0.13	0.70	0.41	0.70	0.41
Curculionidae	*Phyllobius intrusus*	1.60 ± 1.36	1.40 ± 0.24	0.20 ± 0.20	1.4 ± 1.17	0.40 ± 0.40	1,16	2.33	0.15	3.07	0.09	3.07	0.09
Curculionidae	*Phyllobius oblongus*	0.17 ± 0.17	0.33 ± 0.21	2.33 ± 1.17	2.17 ± 0.95	0.17 ± 0.17	1,20	0.16	0.69	8.52	0.01	0.25	0.62
Curculionidae	*Polydrusus formosus*	21.5 ± 9.10 a	22.83 ± 6.01 a	19.83 ± 4.60 a	8.17 ± 5.17 ab	1.33 ± 0.42 b	1,20	2.42	0.14	2.38	0.14	4.16	0.05
Curculionidae	*Dryophthorus americanus*	0 ± 0	0.80 ± 0.20	5.80 ± 3.84	0.60 ± 0.40	1.83 ± 0.31	1,16	0.91	0.36	5.64	0.03	9.95	0.01
Curculionidae	*Anisandrus obesus*	0.20 ± 0.20	0.80 ± 0.58	1.80 ± 0.58	1.40 ± 0.51	1.67 ± 0.33	1,16	0.86	0.37	5.05	0.04	1.78	0.20
Curculionidae	*Anisandrus sayi*	63.5 ± 11.8 ab	93.2 ± 11.2 a	67.0 ± 16.7 ab	39.5 ± 8.45 b	10.17 ± 1.33 c	1,20	0.01	0.94	7.11	0.01	6.31	0.02
Curculionidae	*Hylesinis aculeatus*	22.5 ± 9.27 a	7.50 ± 3.85 ab	6.50 ± 1.57 ab	1.17 ± 0.65 b	10.50 ± 2.57 a	1,20	9.93	0.01	10.70	<0.01	0.46	0.50
Curculionidae	*Orthotomicus caelatus*	0.40 ± 0.24	0 ± 0	1.80 ± 0.58	1.00 ± 0.45	2.20 ± 1.50	1,16	2.96	0.10	11.64	<0.01	0.03	0.87
Curculionidae	*Phloeosinus canadensis*	1.40 ± 0.68	1.20 ± 0.97	0.60 ± 0.40	0.60 ± 0.40	0.40 ± 0.24	1,16	0.02	0.88	2.43	0.14	0.02	0.88
Curculionidae	*Pityokeines sparsus*	0.60 ± 0.40	2.00 ± 0.71	1.80 ± 1.36	5.20 ± 3.62	0.20 ± 0.20	1.16	2.97	0.10	1.86	0.19	0.04	0.85
Curculionidae	*Polygraphus rufipennis*	2.40 ± 0.68	1.80 ± 0.37	0 ± 0	0 ± 0	1.20 ± 0.58	1,16	0.81	0.38	39.85	<0.01	0.81	0.38
Curculionidae	*Xyloterinus politus*	1.67 ± 1.09 bc	1.00 ± 0.52 c	8.50 ± 1.52 a	4.83 ± 1.01 ab	1.83 ± 0.75 bc	1,20	3.61	0.07	32.00	<0.01	0.01	0.93

**Table 4:** Results of generalized linear models testing the effects of horizontal trap position (open vs. edge vs. interior) on species richness and the abundance of Buprestidae, Cerambycidae, and Curculionidae, and sub-taxa, collected in black 12-funnel Lindgren traps in a mixed broadleaf–coniferous forest at Crabbe Mountain, NB, Canada in 2018. All traps were in the upper tree canopy on the edge or interior of the forest or at the equivalent height in the center of a 14–15 m-wide open cut that ran down the mountain (old T-bar line).

Family/Subtaxa	Variable	*F*_2, 27_	*p*	Model
**Buprestidae**	Richness	1.5	0.24	Poisson
	Abundance	4.1	0.03	Negative binomial
**Cerambycidae**	Richness	7.1	<0.01	Gaussian
	Abundance	0.2	0.85	Negative binomial
Lamiinae	Richness	0.6	0.54	Gaussian log
	Abundance	0.3	0.76	Negative binomial
Cerambycinae	Richness	1.1	0.36	Poisson
	Abundance	1.5	0.24	Negative binomial
Lepturinae	Richness	10.2	<0.01	Poisson
	Abundance	16.2	<0.01	Poisson
**Curculionidae**	Richness	3.2	0.06	Poisson
	Abundance	3.4	0.05	Negative binomial
Scolytinae	Richness	4.3	0.03	Poisson
	Abundance	2.4	0.11	Negative binomial
Non-Scolytine	Richness	0.3	0.78	Poisson
Curculionidae	Abundance	2.9	0.07	Negative binomial
**All target taxa**	Richness	6.0	<0.01	Poisson
	Abundance	2.8	0.08	Negative binomial

**Table 5:** Mean catch per trap of species of target taxa captured at Crabbe Mountain, NB, in sufficient numbers for analysis by generalized linear models. Within species, the means followed by different letters were significantly different (Tukey–Kramer’s multiple comparison test on least square means, experiment-wise error = 0.05). Distribution with best fit (lowest corrected Akaike information criterion (AICc)) was the Poisson for 12 species, the negative binomial for 9 species and the Gaussian for 2 species.

Family	Species	Mean Catch per Trap (±SE)					
		Open	Edge	Interior	df	*F*	*p*
Buprestidae	*Dicerca divaricata*	2.30 ± 0.45	2.70 ± 1.01	1.93 ± 0.56	2,27	0.64	0.54
Cerambycidae	*Glycobius speciosus*	2.20 ± 0.49 a	1.30 ± 0.30 ab	0.73 ± 0.22 b	2,27	3.62	0.04
Cerambycidae	*Clytus ruricola*	1.33 ± 0.33	0.37 ± 0.19	0.81 ± 0.28	2,24	2.28	0.12
Cerambycidae	*Cyrtophorus verrucosus*	1.40 ± 0.56	1.96 ± 0.46	1.63 ± 0.42	2,27	0.47	0.63
Cerambycinae	*Anelaphus parallelus*	1.00 ± 0.22	0.43 ± 0.20	1.04 ± 0.54	2,18	0.94	0.41
Cerambycinae	*Anelaphus villosus*	0.63 ± 0.38	0.79 ± 0.37	0.88 ± 0.35	2,21	0.17	0.85
Cerambycinae	*Neoclytus a. acuminatus*	1.00 ± 0.27	1.36 ± 0.56	0.45 ± 0.33	2,21	1.66	0.21
Cerambycinae	*Phymatodes maculicollis*	0.67 ± 0.24	2.11 ± 0.75	1.24 ± 0.56	2,24	1.97	0.24
Cerambycinae	*Xylotrechus colonus*	1.67 ± 0.50	3.23 ± 1.06	0.92 ± 0.57	2,24	2.50	0.10
Cerambycidae	*Aegomorphus modestus*	0.60 ± 0.16	1.54 ± 0.99	0.96 ± 0.27	2,27	0.96	0.40
Cerambycidae	*Astylopsis macula*	5.20 ± 0.73 a	2.62 ± 0.49 b	2.73 ± 0.82 b	2,27	5.84	<0.01
Cerambycidae	*Sternidius rusticus*	1.75 ± 0.45	0.50 ± 0.19	1.79 ± 0.96	2,21	2.80	0.08
Cerambycidae	*Graphisurus fasciatus*	2.89 ± 0.84	2.21 ± 0.52	1.07 ± 0.36	2,24	2.69	0.09
Cerambycidae	*Urgleptes signatus*	19.8 ± 2.09	24.5 ± 7.31	27.6 ± 5.81	2,27	0.68	0.51
Cerambycidae	*Anthophylax cyaneus*	2.33 ± 0.76	0.17 ± 0.17	0 ± 0	2,15	3.25	0.07
Curculionidae	*Acoptus suturalis*	1.00 ± 0.49	2.78 ± 0.74	1.60 ± 0.56	2,27	2.06	0.15
Curculionidae	*Polydrusus cervinus*	15.7 ± 6.03	47.0 ± 27.1	13.7 ± 3.67	2,27	3.26	0.05
Curculionidae	*Phyllobius oblongus*	5.25 ± 2.72 a	1.49 ± 0.62 b	0.75 ± 0.37 b	2,21	10.5	<0.01
Curculionidae	*Polydrusus formosus*	11.4 ± 4.11	9.87 ± 3.65	11.8 ± 4.73	2,27	0.20	0.82
Curculionidae	*Crypturgus borealis*	0.80 ± 0.20 b	2.19 ± 0.28 a	0.53 ± 0.23 b	2,27	15.2	<0.01
Curculionidae	*Anisandrus sayi*	39.6 ± 8.2	50.7 ± 7.2	54.9 ± 8.9	2,27	1.63	0.21
Curculionidae	*Pseudopityophthorus minutissimus*	5.10 ± 1.44 b	14.4 ± 5.53 a	4.32 ± 1.06 b	2,27	6.83	<0.01
Curculionidae	*Xyleborinus attenuatus*	3.22 ± 0.66 a	0.92 ± 0.43 b	0.44 ± 0.24 b	2,24	10.3	<0.01

## Supplementary Materials

The following are available online at https://www.mdpi.com/2075-4450/11/9/573/s1, Table S1: Total number of specimens captured 16 May to 11 September 2014 in 12-funnel black Lindgren traps placed in five different trap positions in/near a mixed broadleaf–coniferous forest near Keswick Ridge, NB. Traps were placed either 30 m inside the forest, or along the edge of the forest that bordered a field, and either in the upper third of the tree canopy (18–25 m height) or in the understory (1.5 m height), or in an open field 30 m from the forest edge (1.5 m height). There were six replicates per trap position. These are raw data on the counts of specimens collected, not standardized for the number of days that each trap was operational., Table S2: Total number of specimens captured 13 June to 6 September 2018 in 12-funnel Lindgren traps placed 30 m inside a forest (Interior) or along its edge, or in the center of a 14–15 m-wide open strip (open) that cut through the forest at Crabbe Mountain, NB. All traps were 12–15 m above the ground. There were ten replicates per trap position, five with green traps and five with black traps. These are raw data, not standardized for the number of days that each trap was operational.
