# Supplementary material for: Impact of Horizontal Edge–Interior and Vertical Canopy–Understory Gradients on the Abundance and Diversity of Bark and Woodboring Beetles in Survey Traps
Source: Insects. 2020 Aug 26;11(9):573. doi: 10.3390/insects11090573 (PMC7564748; doi:10.3390/insects11090573)
Supplement: Supplementary file 1 [file insects-11-00573-s001.pdf]

Table S1. Total number of specimens captured 16 May to 11 September 2014 in 12-funnel black Lindgren traps placed in five different trap positions in/near a mixed broadleaf-coniferous forest near Keswick Ridge, NB. Traps were placed either 30 m inside the forest, or along the edge of the forest that bordered a field, and either in the upper third of the tree canopy (18-25 m height)(Can.) or in the understory (1.5 m height) (Und.), or in an open field 30 m from the forest edge (1.5 m height). There were six traps per trap position. These are raw data on counts of specimens collected, not standardized for number of days that each trap was operational.

Family	Subfamily	Species	Open	Edge		Interior		Total
				Can.	Und.	Can.	Und.	
Buprestidae	Agrilinae	*Agrilus granulatus liragus* Barter & Brown	0	1	0	5	0	6
Buprestidae	Agrilinae	*Brachys aerosus* (Melsheimer)	0	7	0	1	0	8
Buprestidae	Buprestinae	*Chrysobothris scabripennis* Laporte & Gory	0	0	0	1	0	1
Buprestidae	Buprestinae	*Chrysobothris sexsignata* (Say)	0	1	0	0	0	1
Buprestidae	Buprestinae	*Poecilonata cyanipes* (Say)	0	2	0	6	0	8
Buprestidae	Chrysochroinae	*Dicerca divaricata* (Say)	1	17	4	5	0	26
Cerambycidae	Cerambycinae	*Clytus marginicollis* Laporte & Gory	9	8	2	8	0	18
Cerambycidae	Cerambycinae	*Clytus ruricola* (Olivier)	2	10	15	6	7	38
Cerambycidae	Cerambycinae	*Cyrtophorus verrucosus* (Olivier)	10	24	10	6	1	41
Cerambycidae	Cerambycinae	*Microclytus compressicollis* (Castelneau & Gory)	0	4	8	5	15	32
Cerambycidae	Cerambycinae	*Molorchus bimaculatus* Say	1	1	0	2	0	3
Cerambycidae	Cerambycinae	*Neoclytus a. acuminatus* (Fabricius)	1	8	0	7	0	15
Cerambycidae	Cerambycinae	*Neoclytus l. leucozonus* (Castelneau & Gory)	0	1	0	0	0	1
Cerambycidae	Cerambycinae	*Obrium rufulum* Gahan	0	0	0	1	0	1
Cerambycidae	Cerambycinae	*Phymatodes maculicollis* LeConte	17	19	61	16	43	139
Cerambycidae	Cerambycinae	*Phymatodes* sp.	0	0	0	1	0	1
Cerambycidae	Cerambycinae	*Xylotrechus colonus* (Fabricius)	1	0	3	0	2	5
Cerambycidae	Lamiinae	*Aegomorphus modestus* (Gyllenhal)	3	9	0	5	0	14
Cerambycidae	Lamiinae	*Astyeiopus variegatus* (Haldeman)	0	5	0	5	0	10
Cerambycidae	Lamiinae	*Astylopsis macula* (Say)	7	17	44	9	6	76
Cerambycidae	Lamiinae	*Graphisurus fasciatus* (DeGeer)	0	1	1	3	2	7
Cerambycidae	Lamiinae	*Hyperplatys aspersa* (Say)	0	2	0	2	0	4
Cerambycidae	Lamiinae	*Hyperplatys maculata* Haldeman	0	3	1	1	2	7

Cerambycidae	Lamiinae	*Microgoes oculatus* (LeConte)	0	0	1		0	1
Cerambycidae	Lamiinae	*Monochamus scutellatus* (Say)	0	6	9	5	0	20
Cerambycidae	Lamiinae	*Oplosia nubila* (LeConte)	0	0	0	1	0	1
Cerambycidae	Lamiinae	*Pogonocherus pencillatus* LeConte	0	19	1	3	0	23
Cerambycidae	Lamiinae	*Psenocerus supernotatus* (Say)	0	0	0	1	0	1
Cerambycidae	Lamiinae	*Saperda calcarata* Say	0	0	0	1	0	1
Cerambycidae	Lamiinae	*Saperda lateralis* Fabricius	0	0	0	0	1	1
Cerambycidae	Lamiinae	*Sternidius rusticus* (LeConte)	0	2	3	8	0	13
Cerambycidae	Lamiinae	*Urgleptus querc*i (Fitch)	0	1	4	2	4	11
Cerambycidae	Lamiinae	*Urgleptus signatus* (LeConte)	1	12	13	16	19	60
Cerambycidae	Lepturinae	*Analeptura lineola* (Say)	0	0	2	0	0	2
Cerambycidae	Lepturinae	*Anthophylax attenuatus* (Haldeman)	0	0	0	2	0	2
Cerambycidae	Lepturinae	*Anthophylax cyaneus* (Haldeman)	0	1	0	0	0	1
Cerambycidae	Lepturinae	*Bellamira scalaris* (Say)	0	4	1	3	0	8
Cerambycidae	Lepturinae	*Centrodera decolorata* (Harris)	0	7	8	5	7	27
Cerambycidae	Lepturinae	*Evodinus monticola* (Randall)	0	0	6	0	1	7
Cerambycidae	Lepturinae	*Gaurotes cyanipennis* (Say)	0	1	4	0	0	5
Cerambycidae	Lepturinae	*Grammoptera subargentata* (Kirby)	0	0	0	1	0	1
Cerambycidae	Lepturinae	*Lepturobosca chrysocoma* (Kirby)	1	0	0	0	0	0
Cerambycidae	Lepturinae	*Pidonia vibex* (Newman)	0	0	0	0	1	1
Cerambycidae	Lepturinae	*Rhagium inquisitor* (Linnaeus)	0	0	1	1	0	2
Cerambycidae	Lepturinae	*Stictoleptura canadensis (*Olivier)	0	0	0	0	1	1
Cerambycidae	Lepturinae	*Strangalepta abbreviata* (Germar)	0	0	0	0	1	1
Cerambycidae	Lepturinae	*Trachysida mutabilis* (Newman)	0	0	1	0	0	1
Cerambycidae	Lepturinae	*Trigonarthris minnesotana* (Casey)	3	0	2	2	0	4
Cerambycidae	Lepturinae	*Trigonarthris proxima* (Say)	0	2	0	2	0	4
Cerambycidae	Prioninae	*Tragosoma depsarius* (Linnaeus)	2	1	0	5	0	6
Cerambycidae	Spondylidinae	*Atimia c. confusa* (Say)	0	0	0	1	0	1
Cerambycidae	Spondylidinae	*Tetropium cinnamopterum* Kirby	2	0	2	0	11	13
Cerambycidae	Spondylidinae	*Tetropium schwarzianum* Casey	0	0	0	1	1	2
Curculionidae	Baridinae	*Odontocorynus salebrosus* (Casey)	2	0	0	0	0	0
Curculionidae	Conoderinae	*Acoptus suturalis* LeConte	2	7	58	3	4	72
Curculionidae	Cossoninae	*Cossonus platalea* Say	1	0	0	0	0	0

Curculionidae	Cossoninae	*Rhyncolus macrops* Buchanan	0	0	1	0	0	1
Curculionidae	Cossoninae	*Stenoscelis brevis* (Boheman)	0	23	25	12	20	80
Curculionidae	Cryptorhynchinae	*Eubulus bisignatus* (Say)	0	1	0	5	0	6
Curculionidae	Cryptorhynchinae	*Eubulus parochus* (Herbst)	1	2	3	1	0	6
Curculionidae	Curculioninae	*Anthonomus quadrigibbus* (Say)	0	1	3	0	0	4
Curculionidae	Curculioninae	*Dorytomus parvicollis* Casey	0	0	0	4	0	4
Curculionidae	Curculioninae	*Dorytomus* sp. 2	0	0	0	1	0	1
Curculionidae	Curculioninae	*Ellescus ephippiatus* (Say)	0	0	0	3	0	3
Curculionidae	Curculioninae	*Isochnus rufipes* (LeConte)	0	1	2	0	0	3
Curculionidae	Entiminae	*Barypeithes pellucidus* (Boheman)†	0	0	0	0	1	1
Curculionidae	Entiminae	*Phyllobius intrusus* Kono†	2	6	1	7	7	21
Curculionidae	Entiminae	*Phyllobius oblongus* (Linnaeus)†	1	1	14	2	13	30
Curculionidae	Entiminae	*Polydrusus formosus* (Mayer)†	8	125	119	136	49	429
Curculionidae	Entiminae	*Polydrusus impressifrons* (Gyllenhal)†	2	3	4	9	1	17
Curculionidae	Entiminae	*Sciaphilus asperatus* (Bonsdorff)†	0	0	1	0	0	1
Curculionidae	Mesoptiliinae	*Magdalis barbita* (say)	5	0	0	0	0	0
Curculionidae	Molytinae	*Conotrachelus juglandis* LeConte	0	0	1	0	0	1
Curculionidae	Molytinae	*Conotrachelus nenuphar* (Herbst)	5	0	5	0	1	6
Curculionidae	Molytinae	*Hylobius congener* Dalla Torre et al.	0	0	2	0	0	2
Curculionidae	Molytinae	*Pissodes similis* Hopkins	0	0	2	0	1	3
Curculionidae	Scolytinae	*Anisandrus obesus* LeConte	10	1	9	4	7	21
Curculionidae	Scolytinae	*Anisandrus sayi* (Hopkins)	59	355	402	548	231	1536
Curculionidae	Scolytinae	*Cryphalus r. ruficollis* Hopkins	3	0	4	1	4	9
Curculionidae	Scolytinae	*Dendroctonus rufipennis* (Kirby)	0	0	1	0	1	2
Curculionidae	Scolytinae	*Dryocoetes affaber* (Mannerheim)	1	0	0	0	2	2
Curculionidae	Scolytinae	*Dryocoetes autographus* (Ratzeburg)*	0	0	6	0	6	12
Curculionidae	Scolytinae	*Hylastes opacus* Erichson†	0	1	0	0	0	1
Curculionidae	Scolytinae	*Hylastes porculus* Erichson	0	0	1	0	0	1
Curculionidae	Scolytinae	*Hylesinus aculeatus* (Say)	62	121	39	42	7	209
Curculionidae	Scolytinae	*Hylurgopinus rufipes* (Eichhoff)	15	1	0	0	0	1
Curculionidae	Scolytinae	*Ips pini* (Say)	0	0	0	1	0	1
Curculionidae	Scolytinae	*Lymantor decipiens* (LeConte)	0	0	1	0	0	1
Curculionidae	Scolytinae	*Monarthrum mali* (Fitch)	0	0	0	1	0	1
Curculionidae	Scolytinae	*Orthotomicus caelatus* (Eichoff)	11	2	9	0	5	16

Curculionidae	Scolytinae	*Phloeosinus canadensis* Swaine	2	6	3	5	3	17
Curculionidae	Scolytinae	*Pityogenes hopkinsi* Swaine	2	2	2	1	0	5
Curculionidae	Scolytinae	*Pityokeines sparsus* (LeConte)	1	3	9	10	25	47
Curculionidae	Scolytinae	*Polygraphus rufipennis* (Kirby)	6	12	0	9	0	21
Curculionidae	Scolytinae	*Pseudopityophthorus minutissimus* (Zimmermann)	0	0	0	2	0	2
Curculionidae	Scolytinae	*Scolytus piceae* (Swaine)	0	3	0	4	0	7
Curculionidae	Scolytinae	*Scolytus rugulosus* (P.W.J. Müller)†	0	1	0	0	0	1
Curculionidae	Scolytinae	*Trypodendron lineatum* (Olivier)*	0	0	5	2	7	14
Curculionidae	Scolytinae	*Trypophloeus populi* Hopkins	0	3	0	6	0	9
Curculionidae	Scolytinae	*Xyleborinus attenuatus* (Blandford)†	1	0	0	2	1	3
Curculionidae	Scolytinae	*Xyleborinus saxeseni* (Ratzeburg)†	11	2	3	2	1	8
Curculionidae	Scolytinae	*Xyloterinus politus* (Say)	11	8	51	6	29	94
Dryophthoridae	Rhyncophorinae	*Dryophthorus americanus* Bedel	11	0	29	4	3	36

† Non-native species; * Holarctic

Table S2. Total number of specimens captured 13 June to 6 September 2018 in 12-funnel Lindgren traps placed 30 m inside a forest (Interior) or along its edge, or in the center of a 14–15 m wide open strip (Open) that cut through the forest at Crabbe Mountain, NB. All traps were 12–15 m above the ground. There were ten replicates per trap position, five with green traps (Grn) and five with black traps (Blk). These are raw data and have not been standardized for number of days that each trap was operational.

Family	Subfamily	Species	Total number of specimens collected									Grand total
			Open			Edge			Interior			
			Blk	Grn	Total	Blk	Grn	Total	Blk	Grn	Total	
Buprestidae	Agrilinae	*Agrilus anxius* Gory	0	0	0	11	1	12	0	0	0	**12**
Buprestidae	Agrilinae	*Agrilus granulatus liragus* (Barter & Brown)	0	0	0	3	0	3	0	0	0	**3**
Buprestidae	Agrilinae	*Agrilus masculinus* Horn	6	6	12	1	1	2	1	0	1	**15**
Buprestidae	Agrilinae	*Agrilus obsoletoguttatus* Gory	0	3	3	2	1	3	0	0	0	**6**
Buprestidae	Agrilinae	*Agrilus politus* (Say)	1	3	4	2	2	4	1	0	1	**9**
Buprestidae	Agrilinae	*Agrilus ruficollis* (Fabricius)	0	1	1	0	0	0	0	0	0	**1**
Buprestidae	Agrilinae	*Brachys aerosus* (Melsheimer)	0	0	0	1	0	1	0	0	0	**1**
Buprestidae	Buprestinae	*Chrysobothris sexsignata* Say	0	0	0	1	0	1	0	0	0	**1**
Buprestidae	Chrysochroinae	*Dicerca divaricata* (Say)	12	11	23	21	6	27	11	8	19	**69**
Cerambycidae	Cerambycinae	*Anelaphus parallelus* (Newman)	2	5	7	1	2	3	1	6	7	**17**

Cerambycidae	Cerambycinae	*Anelaphus villosus* (Fabricius)	1	4	5	4	2	6	2	5	7	**18**
Cerambycidae	Cerambycinae	*Clytus marginicollis*Laporte & Gory	5	0	5	0	0	0	1	0	1	**6**
Cerambycidae	Cerambycinae	*Clytus ruricola* (Olivier)	4	8	12	3	0	3	4	3	7	**22**
Cerambycidae	Cerambycinae	*Cyrtophorus verrucosus* (Olivier)	6	8	14	12	7	19	6	10	16	**49**
Cerambycidae	Cerambycinae	*Glycobius speciosus* (Say)	10	12	22	7	6	13	3	4	7	**42**
Cerambycidae	Cerambycinae	*Neoclytus a. acuminatus* (Fabricius)	5	3	8	8	2	10	0	3	3	**21**
Cerambycidae	Cerambycinae	*Phymatodes maculicollis* LeConte	5	1	6	4	15	19	3	7	10	**35**
Cerambycidae	Cerambycinae	*Xylotrechus aceris* Fisher	0	0	0	0	1	1	0	0	0	**1**
Cerambycidae	Cerambycinae	*Xylotrechus colonus* (Fabricius)	8	7	15	21	6	27	5	3	8	**50**
Cerambycidae	Cerambycinae	*Xylotrechus quadrimaculatus* (Haldeman)	0	0	0	1	0	1	0	0	0	**1**
Cerambycidae	Cerambycinae	*Xylotrechus undulatus* (Say)	1	0	1	0	0	0	0	0	0	**1**
Cerambycidae	Lamiinae	*Aegomorphus modestus* (Gyllenhal)	2	4	6	10	3	13	4	5	9	**28**
Cerambycidae	Lamiinae	*Astyleiopus variegatus* (Haldeman)	1	1	2	0	3	3	0	2	2	**7**
Cerambycidae	Lamiinae	*Astylopsis macula* (Say)	29	23	52	16	9	25	20	7	27	**104**
Cerambycidae	Lamiinae	*Graphisurus fasciatus* (DeGeer)	12	14	26	11	8	19	4	5	9	**54**
Cerambycidae	Lamiinae	*Hyperplatys maculata* Haldeman	0	1	1	0	2	2	3	2	5	**8**
Cerambycidae	Lamiinae	*Microgoes oculatus* (LeConte)	0	0	0	0	0	0	2	1	3	**3**
Cerambycidae	Lamiinae	*Monochamus s. scutellatus* (Say)	1	0	1	0	0	0	0	2	2	**3**
Cerambycidae	Lamiinae	*Pogonocherus penicillatus* LeConte	0	0	0	0	0	0	0	6	6	**6**
Cerambycidae	Lamiinae	*Psenocerus supernotatus* (Say)	1	1	2	3	0	3	0	5	5	**10**
Cerambycidae	Lamiinae	*Saperda calcarata* Say	0	0	0	1	0	1	0	0	0	**1**
Cerambycidae	Lamiinae	*Sternidius rusticus* (LeConte)	7	7	14	2	2	4	4	10	14	**32**
Cerambycidae	Lamiinae	*Tetrops praeusta* (Linnaeus)	0	0	0	1	0	1	0	0	0	**1**
Cerambycidae	Lamiinae	*Urgleptes querci* (Fitch)	0	1	1	1	3	4	1	1	2	**7**
Cerambycidae	Lamiinae	*Urgleptes signatus* (LeConte)	99	99	198	114	127	241	139	134	273	**712**
Cerambycidae	Lepturinae	*Anoplodera pubera* (Say)	3	0	3	0	0	0	0	0	0	**3**
Cerambycidae	Lepturinae	*Anthophylax cyaneus* (Haldeman)	6	8	14	1	0	1	0	0	0	**15**
Cerambycidae	Lepturinae	*Anthophylax viridis* LeConte	3	2	5	1	2	3	0	0	0	**8**
Cerambycidae	Lepturinae	*Bellamira scalaris* (Say)	3	1	4	1	0	1	0	0	0	**5**
Cerambycidae	Lepturinae	*Evodinus monticola* (Randall)	1	1	2	3	0	3	0	0	0	**5**
Cerambycidae	Lepturinae	*Grammoptera subargentata* (Kirby)	0	1	1	0	0	0	0	0	0	**1**
Cerambycidae	Lepturinae	*Neoalosterna capitata* (Newman)	1	0	1	0	0	0	0	0	0	**1**

Cerambycidae	Lepturinae	*Pidonia ruficollis* (Say)	3	0	3	1	0	1	1	0	1	**5**
Cerambycidae	Lepturinae	*Pidonia vibex* (Newman)	1	0	1	0	0	0	0	0	0	**1**
Cerambycidae	Lepturinae	*Stictoleptura canadensis* (Olivier)	3	0	3	0	0	0	0	0	0	**3**
Cerambycidae	Lepturinae	*Strangalepta abbreviata* (Germar)	1	1	2	1	0	1	0	0	0	**3**
Cerambycidae	Lepturinae	*Trachysida mutabilis* (Newman)	2	5	7	1	3	4	0	0	0	**11**
Cerambycidae	Lepturinae	*Trigonarthris minnesotana* (Casey)	1	1	2	0	0	0	0	1	1	**3**
Cerambycidae	Lepturinae	*Trigonarthris proxima* (Say)	1	0	1	0	0	0	0	0	0	**1**
Cerambycidae	Spondylidinae	*Tetropium cinnamopterum* Kirby	0	0	0	1	0	1	1	0	1	**2**
Curculionidae	Conoderinae	*Acoptus suturalis* LeConte	3	7	10	16	10	26	6	10	16	**52**
Curculionidae	Conoderinae	*Psomus armatus* Dietz	0	0	0	1	0	1	0	0	0	**1**
Curculionidae	Cossoninae	*Himatium errans* LeConte	0	0	0	0	0	0	1	0	1	**1**
Curculionidae	Cossoninae	*Rhyncolus minor* (Horn)	0	0	0	0	0	0	1	0	1	**1**
Curculionidae	Cossoninae	*Phloeophagus apionides* Horn	0	0	0	2	1	3	1	1	2	**5**
Curculionidae	Cossoninae	*Phloeophagus canadensis* Van Dyke	1	0	1	3	0	3	1	0	1	**5**
Curculionidae	Curculioninae	*Anthonomus* sp. Germar	0	0	0	2	0	2	1	1	2	**4**
Curculionidae	Curculioninae	*Curculio obtusus* (Blanchard)	1	2	3	0	0	0	0	0	0	**3**
Curculionidae	Curculioninae	*Curculio sp.* Linnaeus	1	0	1	0	0	0	0	0	0	**1**
Curculionidae	Curculioninae	*Lignyodes horridulus* (Casey)	0	1	1	0	0	0	0	0	0	**1**
Curculionidae	Curculioninae	*Orchestes sp.* Illiger	1	0	1	4	3	7	1	0	1	**9**
Curculionidae	Curculioninae	*Piazorhinus scutellaris* (Say)	3	2	5	2	2	4	2	2	4	**13**
Curculionidae	Curculioninae	*Pseudanthonomus validus* Dietz	0	0	0	1	0	1	0	0	0	**1**
Curculionidae	Curculioninae	*Tychius picirostris* (Fabricius)	0	1	1	1	1	2	1	2	3	**6**
Curculionidae	Dryophthorinae	*Dryophthorus americanus* Bedel	1	0	1	0	0	0	0	1	1	**2**
Curculionidae	Entiminae	*Barypeithes pellucidus* (Boheman)†	0	0	0	1	0	1	0	0	0	**1**
Curculionidae	Entiminae	*Phyllobius intrusus* Kono†	0	3	3	0	1	1	5	3	8	**12**
Curculionidae	Entiminae	*Phyllobius oblongus* (Linnaeus)†	29	5	34	5	6	11	4	2	6	**51**
Curculionidae	Entiminae	*Polydrusus cervinus* (Linnaeus)†	102	55	157	278	128	406	73	63	136	**699**
Curculionidae	Entiminae	*Polydrusus formosus* (Mayer)†	35	79	114	40	51	91	53	64	117	**322**
Curculionidae	Molytinae	*Conotrachelus nenuphar (Herbst)*	0	1	1	1	0	1	0	0	0	**2**
Curculionidae	Molytinae	*Conotrachelus posticatus* Boheman	0	1	1	0	0	0	0	0	0	**1**
Curculionidae	Molytinae	*Magdalis hispoides* LeConte	0	0	0	0	1	1	0	1	1	**2**

Curculionidae	Molytinae	*Magdalis sp.* Germar	0	1	1	0	0	0	0	0	0	**1**
Curculionidae	Curculioninae	*Tachyerges niger* (Horn)	0	0	0	1	0	1	0	0	0	**1**
Curculionidae	Scolytinae	*Anisandrus obesus* (LeConte)	6	2	8	4	1	5	3	0	3	**16**
Curculionidae	Scolytinae	*Anisandrus sayi* (Hopkins)	269	127	396	282	211	493	338	191	529	**1418**
Curculionidae	Scolytinae	*Cryphalus ruficollis* Hopkins	0	0	0	1	1	2	1	0	1	**3**
Curculionidae	Scolytinae	*Crypturgus borealis* Swaine	6	2	8	13	8	21	0	5	5	**34**
Curculionidae	Scolytinae	*Dryocoetes affaber* (Mannerhiem)	0	0	0	2	2	4	0	0	0	**4**
Curculionidae	Scolytinae	*Dryocoetes autographus* (Ratzeburg)	4	0	4	3	0	3	0	0	0	**7**
Curculionidae	Scolytinae	*Gnathotrichus materiarius* (Fitch)	0	0	0	7	5	12	0	0	0	**12**
Curculionidae	Scolytinae	*Hylastinus obscurus* (Marsham)†	1	0	1	0	1	1	0	0	0	**2**
Curculionidae	Scolytinae	*Hylesinus aculeatus* Say	0	7	7	0	0	0	0	0	0	**7**
Curculionidae	Scolytinae	*Lymantor decipiens* (LeConte)	1	0	1	0	0	0	0	0	0	**1**
Curculionidae	Scolytinae	*Monarthrum mali* (Fitch)	4	1	5	9	2	11	1	1	2	**18**
Curculionidae	Scolytinae	*Pityogenes hopkinsi* Swaine	0	0	0	0	0	0	0	1	1	**1**
Curculionidae	Scolytinae	*Pityokteines sparsus* (LeConte)	0	2	2	1	0	1	0	0	0	**3**
Curculionidae	Scolytinae	*Pityophthorus spp.*Eichhoff	0	0	0	0	0	0	1	0	1	**1**
Curculionidae	Scolytinae	*Polygraphus rufipennis* (Kirby)	3	0	3	2	1	3	1	1	2	**8**
Curculionidae	Scolytinae	*Pseudopityophthorus minutissimus* (Zimmermann)	27	24	51	93	50	143	21	21	42	**236**
Curculionidae	Scolytinae	*Scolytus piceae* (Swaine)	2	3	5	2	1	3	0	7	7	**15**
Curculionidae	Scolytinae	*Trypodendron lineatum* (Olivier)*	0	1	1	1	0	1	0	0	0	**2**
Curculionidae	Scolytinae	*Xyleborinus attenuatus* (Blandford)†	15	14	29	7	1	8	4	0	4	**41**
Curculionidae	Scolytinae	*Xyleborinus saxesenii* (Ratzeburg)†	1	0	1	1	1	2	0	0	0	**3**
Curculionidae	Scolytinae	*Xyloterinus politus* (Say)	1	1	2	3	1	4	0	0	0	**6**

† non-native; * holartic
